# *ZNF518B* gene up-regulation promotes dissemination of tumour cells and is governed by epigenetic mechanisms in colorectal cancer

**DOI:** 10.1038/s41598-019-45411-9

**Published:** 2019-06-27

**Authors:** Francisco Gimeno-Valiente, Ángela L. Riffo-Campos, Azahara Vallet-Sánchez, Sofía Siscar-Lewin, Valentina Gambardella, Noelia Tarazona, Andrés Cervantes, Luis Franco, Josefa Castillo, Gerardo López-Rodas

**Affiliations:** 1 Institute of Health Research, INCLIVA, Valencia, Spain; 2 Present Address: Centro De Excelencia de Modelación y Computación Científica, Departamento de Anatomía Patológica, Universidad de La Frontera, Temuco, Chile; 3 Present Address: Department of Cell Biology, Universitat de València, Burjassot, Valencia, Spain; 4 Present Address: Department of Microbial Pathogenicity Mechanisms, Hans-Knoell-Institute, Jena, Germany; 5 Centro de Investigación Biomédica en Red en Cáncer (CIBERONC), Madrid, Spain; 6 Department of Medical Oncology, University Hospital, Universitat de València, Valencia, Spain; 7 Department of Biochemistry and Molecular Biology, Universitat de València, Valencia, Spain

**Keywords:** Epigenetics, Colorectal cancer

## Abstract

Most of colorectal cancer CRC-related death is due to metastasis and the finding of markers for prognosis of invasiveness, constitutes an appealing challenge. Here, after analysing cDNA array containing 43 tumour and 5 normal mucosa samples, we report that the expression of the *ZNF518B* gene as a whole and that of its two major splicing isoforms are significantly increased in tumours. The canonical isoform was also up-regulated in a patients’ cohort containing 70 tumour and 69 adjacent tissue samples. The effects of silencing *ZNF518B* on the phenotype of CRC cell lines were then studied. The gene does not affect cell proliferation, but plays a significant role in cell migration and invasiveness and induces changes in the epithelial-to-mesenchymal transition markers, suggesting that *ZNF518B* favours tumour cell dissemination. To study the regulation of the gene, transcription-related changes in nucleosomal organisation and epigenetic marks around the transcriptional start site were analysed. The positioning of a nucleosome over the transcription start site and the differential presence of the epigenetic marks H3K9ac, H3K27ac, H3K4me3 and H3K9me3 correlate with gene expression. Inhibition of histone deacetylases increases the transcription of *ZNF518B*, which may be a candidate for invasiveness prognosis in CRC and a target for epigenetic drugs.

## Introduction

Colorectal cancer (CRC) ranks among the most prevalent carcinomas worldwide and 1.4 million new cases are diagnosed each year^1^. Although precision medicine has allowed effectively treating and curing many types of CRC, the long-term survival rate of patients with advanced metastatic disease is still very poor. The disease causes 700,000 deaths per annum, most of them related to metastatic spread^2^. In spite of the attempts carried out in the last years to establish a molecular signature for development and dissemination of CRC, there are still many unsolved questions. In this context, any effort to increase our knowledge on the key factors involved in the progression of CRC would be welcome.

We have reported that *ZNF518B* gene is differentially expressed in some human CRC cell lines^3^, but the knowledge on the role and properties of the gene is very limited. According to the data retrieved from the Ensembl genome browser (www.ensembl.org), human *ZNF518B* locus maps to chromosome 4 (10,439,874–10,457,410) and is transcribed from the reverse strand. The gene is not expressed in Caco2, RKO or SW48 cells, while its transcription could be detected in HCT116, DLD1, D-Mut1 and DWT7m cell lines^3^. Alternative splicing gives rise to five isoforms. Isoforms 1 and 2 are the major ones and the abundance of their mRNAs is roughly similar in the cell lines expressing the gene^3^. The canonical isoform 1 encodes a protein 1074 residues long, while isoform 2 includes an open reading frame which may putatively produce a truncated form of the N-terminus of the protein. No evidence for the actual existence of this truncated protein exists. The three remaining isoforms are non-protein coding.

Apart from the data mentioned above, there are only a few reports in the literature concerning the role of *ZNF518B*. It is clear from sequence data that isoform 1 encodes a zinc-finger protein, a putative transcriptional factor for which the identification of its possible targets remains to be determined. Single nucleotide polymorphisms have been described to occur at or close the *ZNF518B* gene in association with gout and/or serum urate concentration^4–6^. More interestingly to our purpose, in a proteomic analysis of the interactome of G9A, Maier *et al*. found that ZNF518B is one of the partners of the histone methyltransferase. The whole *in vitro* translated ZNF518B protein, as well as its deletion fragments, are able to interact with G9A, showing that multiple domains are involved in the interaction. ZNF518B positively regulates the methylation of H3K9, suggesting that its binding to G9A result in the activation of the enzyme^7^.

These facts establish a potential relationship between *ZNF518B* and cancer, because a dysregulation of G9A has been found in several types of cancer^8^. Specifically, the enzyme is overexpressed in CRC tissues when compared to paired normal epithelium and its down-regulation inhibits proliferation of cancer cells^9^. In view of the above data, it seems clear that an overexpression of *ZNF518B* may have deleterious effects on cancer progression and invasiveness. However, to date no reports on the differential expression of *ZNF518B* between cancerous and normal tissues have appeared. Should the gene be overexpressed in cancer tissues, it might be added to the available panel of molecular markers. In the present research, we first analysed the level of *ZNF518B* in 45 CRC patient-derived cDNA samples, to find that there is a significantly higher expression in these samples than in normal mucosa. Isoform 1 is also up-regulated in a larger patients’ cohort. In view of these results, we further describe in this paper that *ZNF518B* is involved in migration and invasiveness of CRC cell lines, and that the regulation of gene expression takes place at a chromatin level.

## Results

### *ZNF518B* is overexpressed in human CRC

**Figure 1:**
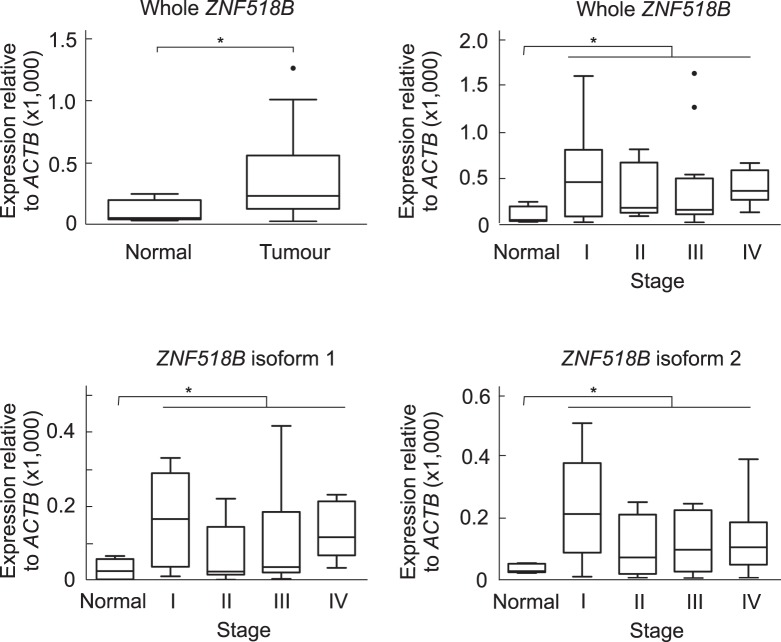
*ZNF518B* is overexpressed in CRC patients. The figure shows box plots with whiskers with maximum 1.5 IQR of the *ZNF518B* expression measured by qPCR in an OriGene cDNA array of normal and tumour samples. The plots correspond to the comparison of whole gene and its major isoforms in normal *versus* all tumour tissues or stage-classified tumour tissues. Results were compared with the Kruskal-Wallis test. (*p < 0.05).

**Figure 2:**
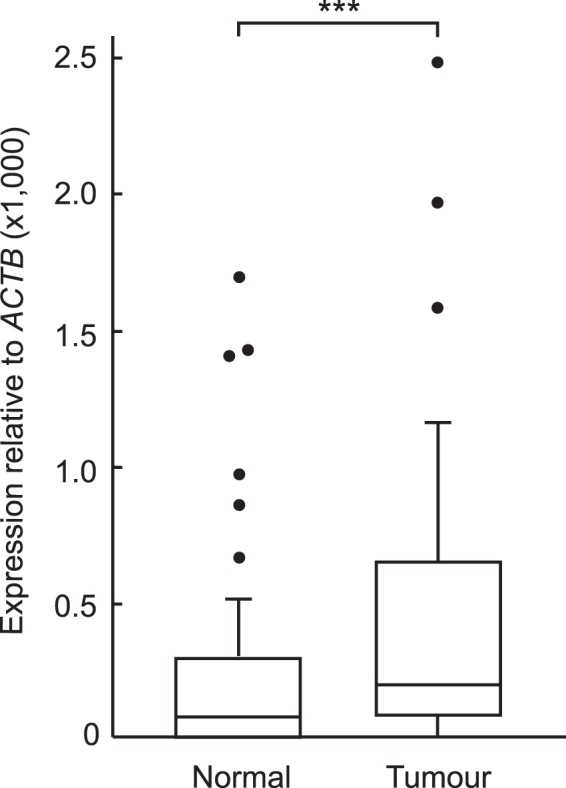
Overexpression of the *ZNF518B* canonical isoform 1 in a prospective cohort of CRC patients from our University Hospital (70 tumour and 69 non-tumour adjacent samples). The figure shows box plots with whiskers as in Fig. 1, including the position of medians. Results were compared with the Mann-Whitney test. (***p < 0.001).

The *ZNF518B* expression analysis in human CRC patients was first carried out with a TissueScan cDNA array (OriGene). The array used contained 5 cDNA samples from normal mucosa; the analysis of the original tissues, provided by the supplier, showed normal histological appearance. The array also contained 43 cDNA samples from CRC patients, 10 from stage I, 13 from stage II, 14 from stage III and 6 from stage IV. They corresponded to 21 males and 27 females, with an age range from 36 to 92 years and an average of 68.5 ± 13.3 years. The expression of the *ZNF518B* gene as a whole and of its isoforms 1 and 2 was determined by RT-qPCR in all the samples. Both the whole gene and its isoforms are significantly overexpressed in human CRC at all stages but no significant differences were observed from one stage to another (Fig. 1). Then, the expression of the gene was analysed in a prospective study with a cohort of 101 patients, who underwent surgery at the University Hospital (Valencia) in the last 3 years (Supplementary Table S1). The quality of the cDNA prepared by retrotranscription of RNA isolated from paraffin-embedded samples, together with the low expression level of the gene, did not allow us to amplify all the samples by RT-qPCR. Therefore, only the results obtained with the canonical isoform from non-paired 70 tumour and 69 non-tumour adjacent samples are presented in Fig. 2, which shows that, in agreement with the data obtained from the cDNA array, *ZNF518B* is significantly up-regulated in human CRC.

Once determined that the expression of the gene is enhanced in CRC tissues, the mechanisms putatively involved in the *ZNF518B*-related malignancy development, as well as those governing the regulation of the gene expression, were studied *in vitro* with CRC-derived cell lines. The results are described in the next sections.

### Effects of *ZNF518B* on the proliferation of CRC cells

To explore the function of *ZNF518B* in human CRC progression, the effects of the gene on cell growth were first analysed. To do this, the gene was silenced with *ZNF518B* siRNA. After checking the effects of several commercial siRNAs on the knocking-down of both major isoforms of the gene, the equimolar mixture of the two siRNAs described under Materials and Methods was further used. One of the siRNAs silenced both isoforms 1 and 2, while the other targets only at isoform 1 (Supplementary Fig. S1a).

**Figure 3:**
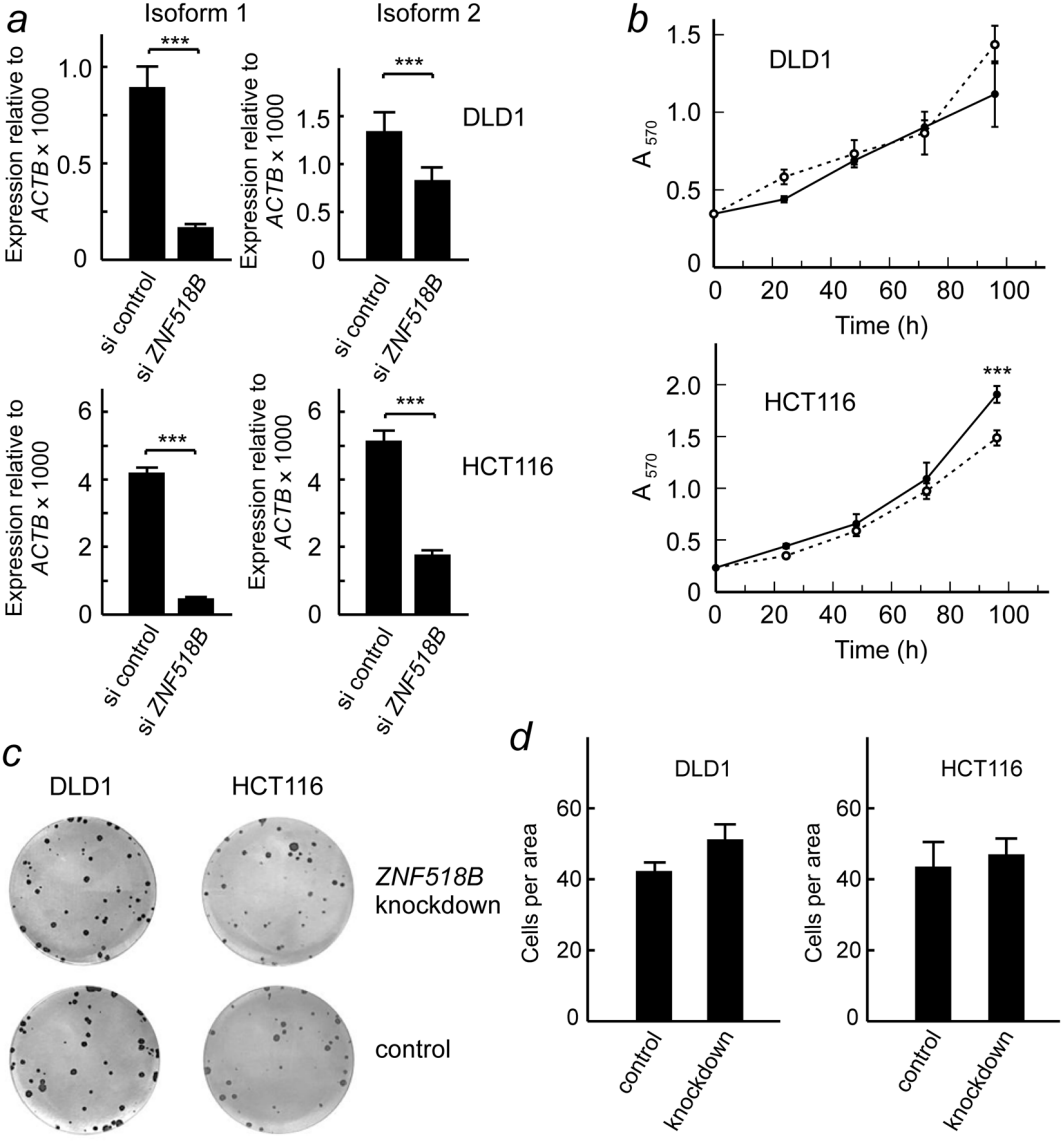
Effects of *ZNF518B* knocking-down on the proliferation of DLD1 and HCT116 cells. (**a**) Efficiency of knocking-down for isoforms 1 and 2 in DLD1 and HCT116 cells after 48 h of transfection. (**b**) Influence of knocking-down on cell proliferation as measured by MTT assays; continuous lines, control cells; broken lines, cells transfected with *ZNF518B* siRNA. (**c**) Effects of *ZNF518B* knocking-down in a representative colony formation assay. (**d**) Quantification of the different cell colony formation assays. Statistical analysis of data in **a** and **d** was carried out with the Mann-Whitney test, whereas two-way ANOVA was used for data in panel b. (***p < 0.001).

To achieve a most effective knocking down, in most of the following experiments we used an equimolar mixture of both siRNAs. As shown in Fig. 3a, the mixed siRNAs are more efficient in knocking-down the expression of isoform 1 (to 18% in DLD1 and to 12% in HCT116 cells) than that of isoform 2 (to 62% in DLD1 and to 34% in HCT116) 48 h after transfection. These trends are maintained after 96 h (Supplementary Fig. S2). The consequences of the knocking-down were evaluated by both, MTT and colony formation assays. MTT assay showed that *ZNF518B* does not appreciably influence proliferation of DLD1, while a decrease in the expression of the gene causes a small, but significant, reduction in the proliferation of HCT116 cells (Fig. 3b). Cell cycle analysis revealed no significant differences in any of the cell lines after *ZNF518B* knocking-down (Supplementary Fig. S3). Results of Fig. 3c,d show that *ZNF518B* does not appreciably influence the colony-forming capacity of DLD1 and HCT116 cells.

### Involvement of *ZNF518B* in epithelial-to-mesenchymal transition

**Figure 4:**
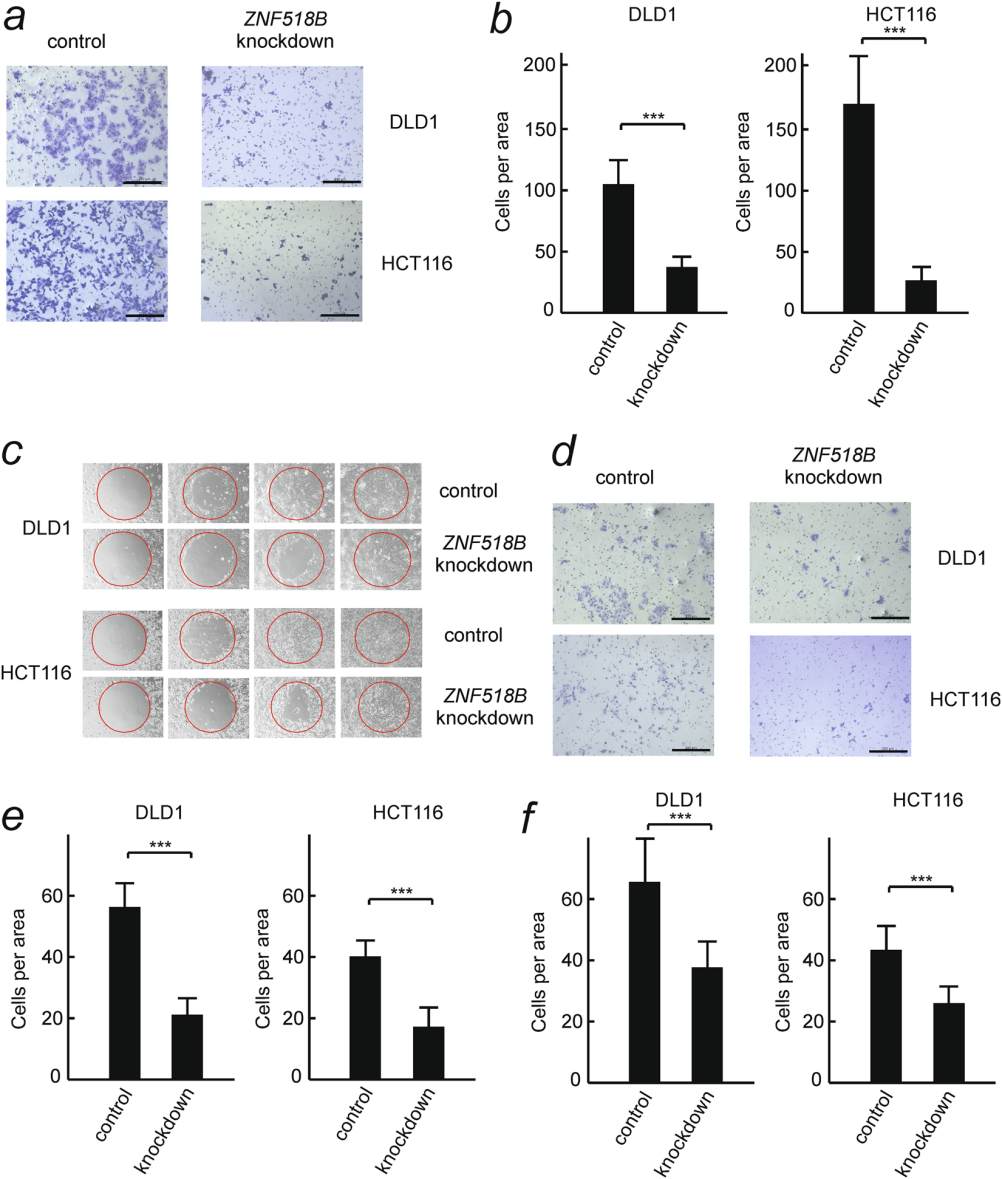
Effects of *ZNF518B* knocking-down on the migration and invasiveness of DLD1 and HCT116 cells. (**a**,**b**) Representative images and quantitative analysis showing the suppression of migration of DLD1 and HCT116 cells by knocking-down the *ZNF518B* gene. The assay was done in transwell chambers. (**c**) Wound-healing assay showing the diminution of cell migration after silencing the *ZNF518B* gene. (**d**,**e**) Representative images and quantitative analysis showing the suppression of invasiveness of DLD1 and HCT116 cells by knocking-down the *ZNF518B* gene. The assay was carried out in Matrigel-coated transwell chambers. (**f**) Quantification of cell-adhesion assays to collagen type I-coated dishes, showing the loss of adhesiveness after silencing the *ZNF518B* gene. Statistical analysis was carried out with the Mann-Whitney test. (***p < 0.001).

**Figure 5:**
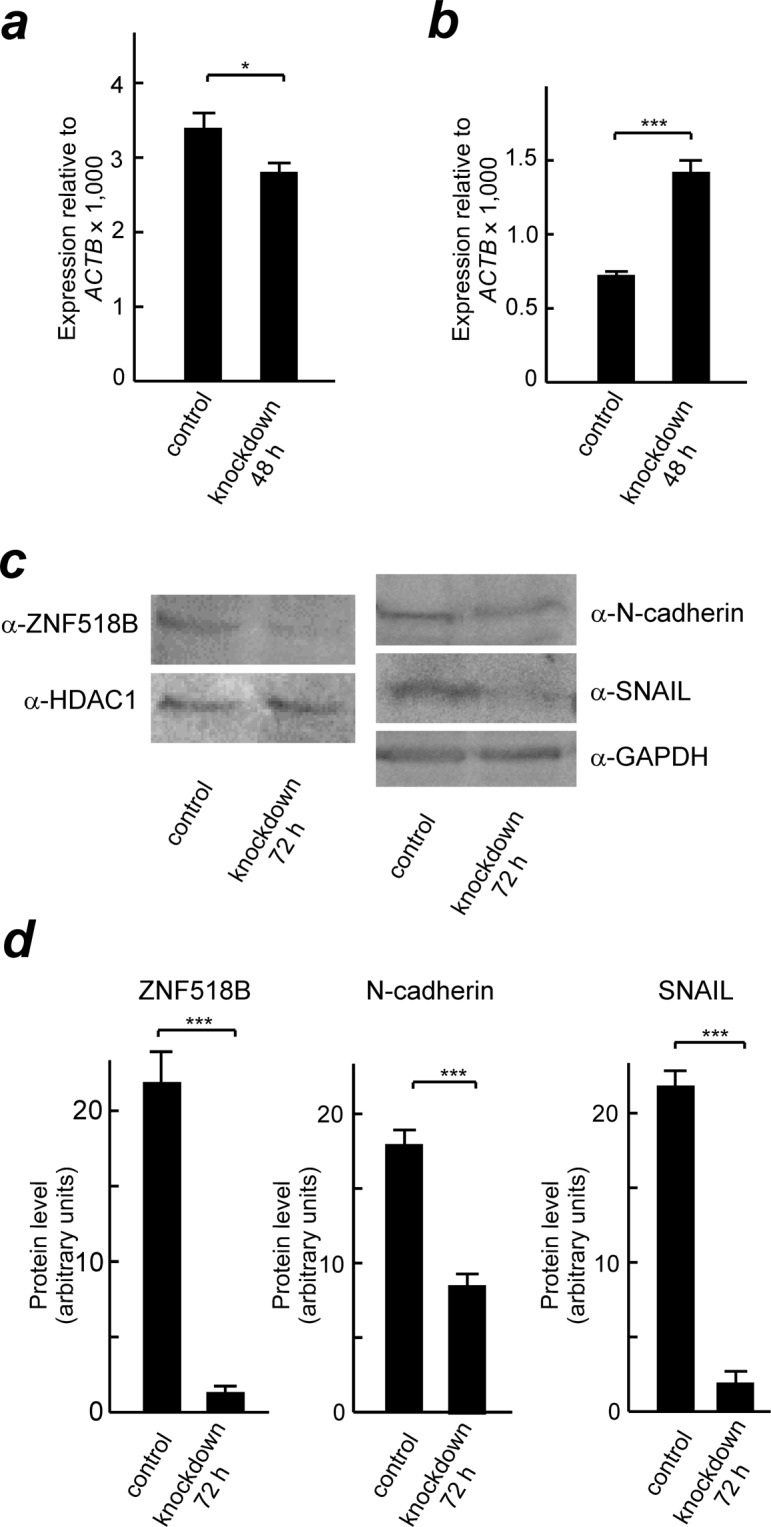
Effects of *ZNF518B* expression on the level of EMT markers. DLD1 cells were treated with mixed *ZNF518B* siRNAs or with scrambled siRNA (control) for 48 and 72 h and the EMT markers were analysed. The levels of the *SNAI1* (**a**) and *CHD1* (**b**) genes were determined by RT-qPCR. (**c**) Western blots showing the level of ZNF518B (left) and N-cadherin and SNAIL (right) after 72 h of silencing *ZNF518B*. Histone deacetylase 1 (HDAC) or glyceraldehyde-3-phosphate dehydrogenase (GAPDH) were used as loading controls. Due to the different size of ZNF518B and of the EMT markers, the electrophoreses were carried out separately in 6% and 12% polyacrylamide gels, respectively. Ponceau-stained membranes were cropped according to the molecular weight of the corresponding proteins and the resulting strips were developed with the antibodies described under Materials and Methods. (**d**) ImageJ analysis of the western blots of panel ***c***. Four grey values relative to the loading controls were measured in every case and averaged. Statistical analysis was carried out with the Mann-Whitney test. (*p < 0.05; ***p < 0.001).

The effects of silencing *ZNF518B* (see above) on cell migration were studied with DLD1 and HCT116 cell lines by transwell and wound healing assays. The results of the transwell analysis (Fig. 4a,b) show that *ZNF518B* knocking-down significantly reduces cell migration and the wound healing assay (Fig. 4c) confirmed these results. The migration assays were also done with the individual siRNAs and in both cases a significant reduction of cell migration after knocking down the gene was observed (Supplementary Fig. S4). These results show that the two siRNAs do not have off-target effects. When the transwell analysis was carried out through a Matrigel layer to explore the effects of the gene on invasiveness, it was observed that silencing of the gene significantly reduces the invasion of cells through extracellular matrix (Fig. 4d,e). Finally, the adhesion of cells to type I collagen fibres was also influenced by silencing *ZNF518B* (Fig. 4f). All of the above events are usually associated with epithelial-to-mesenchymal transition (EMT). Therefore, the effects of *ZNF518B* expression on the level of EMT markers were next examined. Figure 5a shows that knocking-down *ZNF518B* in DLD1 cells resulted in a significant decrease in the expression of *SNAI1*gene after treating the cells with siRNA for 48 h. SNAIL, the product of this gene, is a transcriptional repressor of the E-cadherin gene and, accordingly, *CHD1* transcription is significantly increased in DLD1 cells after knocking-down the *ZNF518B* gene (Fig. 5b). The levels of the proteins encoded by these genes were determined after 72 h of treatment with siRNA, taking into account the lag time existing between transcription and steady state accumulation of protein. SNAIL was drastically reduced by silencing *ZNF518B* (Fig. 5c,d). The level of E-cadherin protein was so low that we could not detect it. This was not the case with N-cadherin which decreases after silencing. The observed decrease in the levels of SNAIL and N-cadherin parallels that of ZNF518B protein (Fig. 5c,d). From these experiments it can be concluded that *ZNF518B* promotes migration and invasiveness of CRC cells. Therefore, the gene might be considered as involved in EMT and cancer cell dissemination, probably through type I collagen fibres.

### Factors influencing *ZNF518B* expression

In view of the above results, it is interesting to explore the molecular causes involved in the regulation of *ZNF518B* expression. This may be adequately done through a comparison of the behaviour of SW48 and DLD1, because the gene is not transcribed in the former cell line, while it is in the second one.

**Figure 6:**
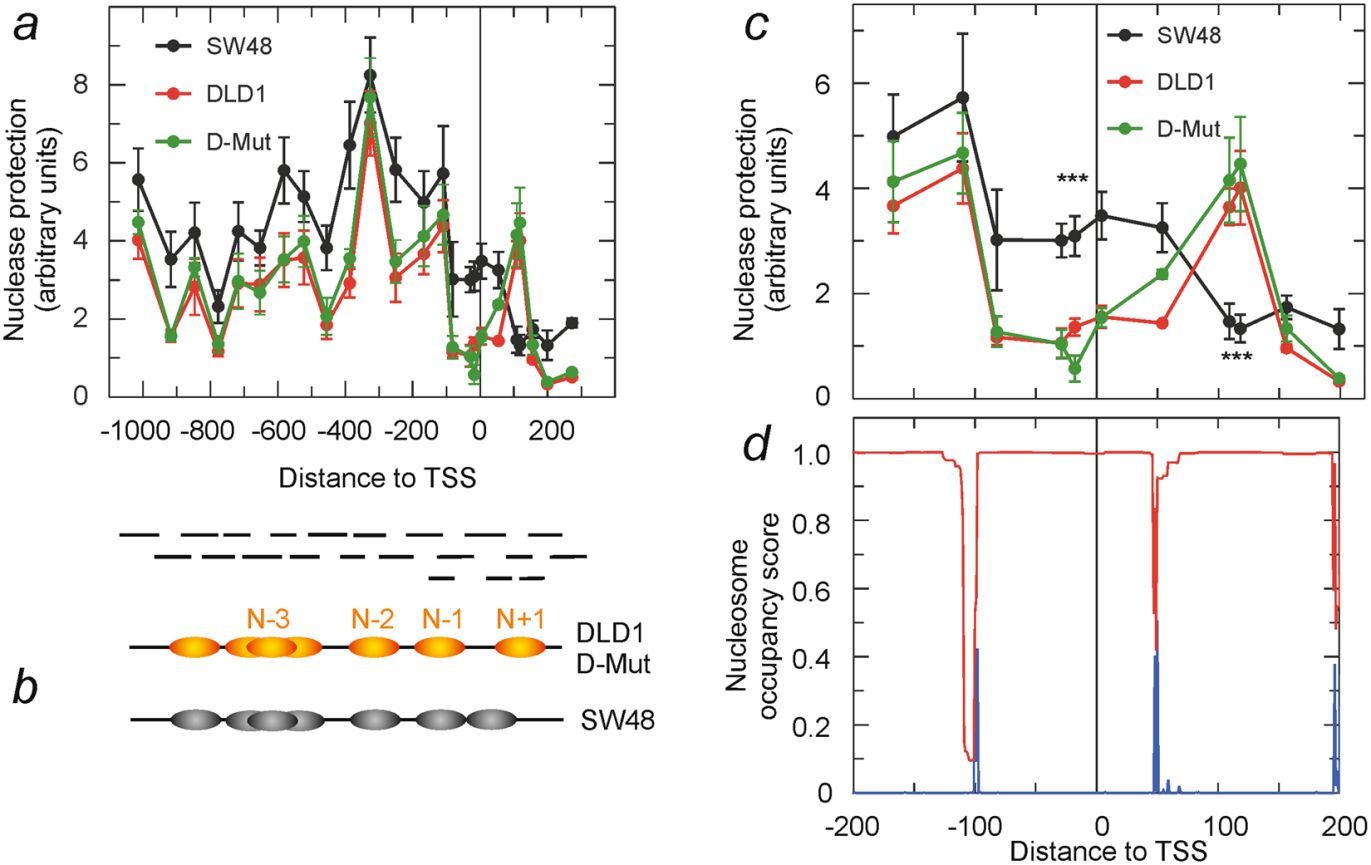
Organisation of chromatin at the promoter and proximal transcribed region of *ZNF518B* gene. The experiments were carried out with DLD1, SW48 and D-Mut1 cells. (**a**) Micrococcal nuclease protection assay. The protection, determined as described in the text, is plotted against the position of the centre of the tiled amplicons used, whose location is given below. The coordinates are given in bp relative to TSS. (**b**) Model for nucleosome positioning. (**c**) Detail of the nuclease protection assay in the region from −200 to +200. (**d**) Prediction of the nucleosome occupancy score (*red*) and of the probability of starting nucleosomes (*blue*), as determined by the NuPoP program (12).

As nucleosome organisation may play a regulatory role in gene expression, the chromatin structure at the promoter and proximal transcribed region was first studied. The results of a micrococcal nuclease protection assay from −1,000 to +300 relative to the transcription start site (TSS) are given in Fig. 6a, which also includes the location of the tiled amplicons used (see Supplementary Table S2). The protection profile of the D-Mut1 cell line, in which the *ZNF518B* gene is also expressed^3^, was included as a control. This assay offers a convenient method to analyse nucleosome positioning^10,11^. Most of the peaks can be ascribed to the presence of nucleosomes as their width is compatible with nucleosomal size. A model for the nucleosomal organisation of the region under study is depicted in Fig. 6b. The nuclease-protected region between −500 and −800 may be ascribed either to two nucleosomes closely packed together or to a single nucleosome, N − 3, which may occupy a series of sites due to sequence-directed, rotational positioning determinants. At any rate, as this aspect of chromatin structure is similar in the three cell lines, choosing one possibility or another is immaterial to the question of transcriptional regulation of the gene.

When examining the entire region analysed (Fig. 6a) it can be observed that the chromatin organisation in the three cell lines is very similar save in two details. First, the overall protection to micrococcal nuclease is higher in the SW48 cell line, in which *ZNF518B* is not expressed. Second, there is a clear difference in the chromatin structure of the cell lines around the TSS. While in DLD1 and D-Mut1 cells the location of five nucleosomes (N + 1 and N − 4 to N − 1) may be postulated and the TSS encompasses a nucleosome-free region, in SW48 cells the TSS is protected, suggesting that an additional nucleosome is positioned over it. This region is observed in more detail in Fig. 6c. The sequence-based analysis of nucleosome positioning recovered from the NuPop programme (Fig. 6d) predicts the presence of a nucleosome between −100 and +45. In spite of the high probability predicted, the DLD1 and D-Mut1 cells lack a nucleosome over TSS, while the nuclease protection assay suggested that a nucleosome actually is located in that position in SW48 cells. It seems obvious that the chromatin structure accounted, at least in part, for the differences in gene expression between these cell lines.

**Figure 7:**
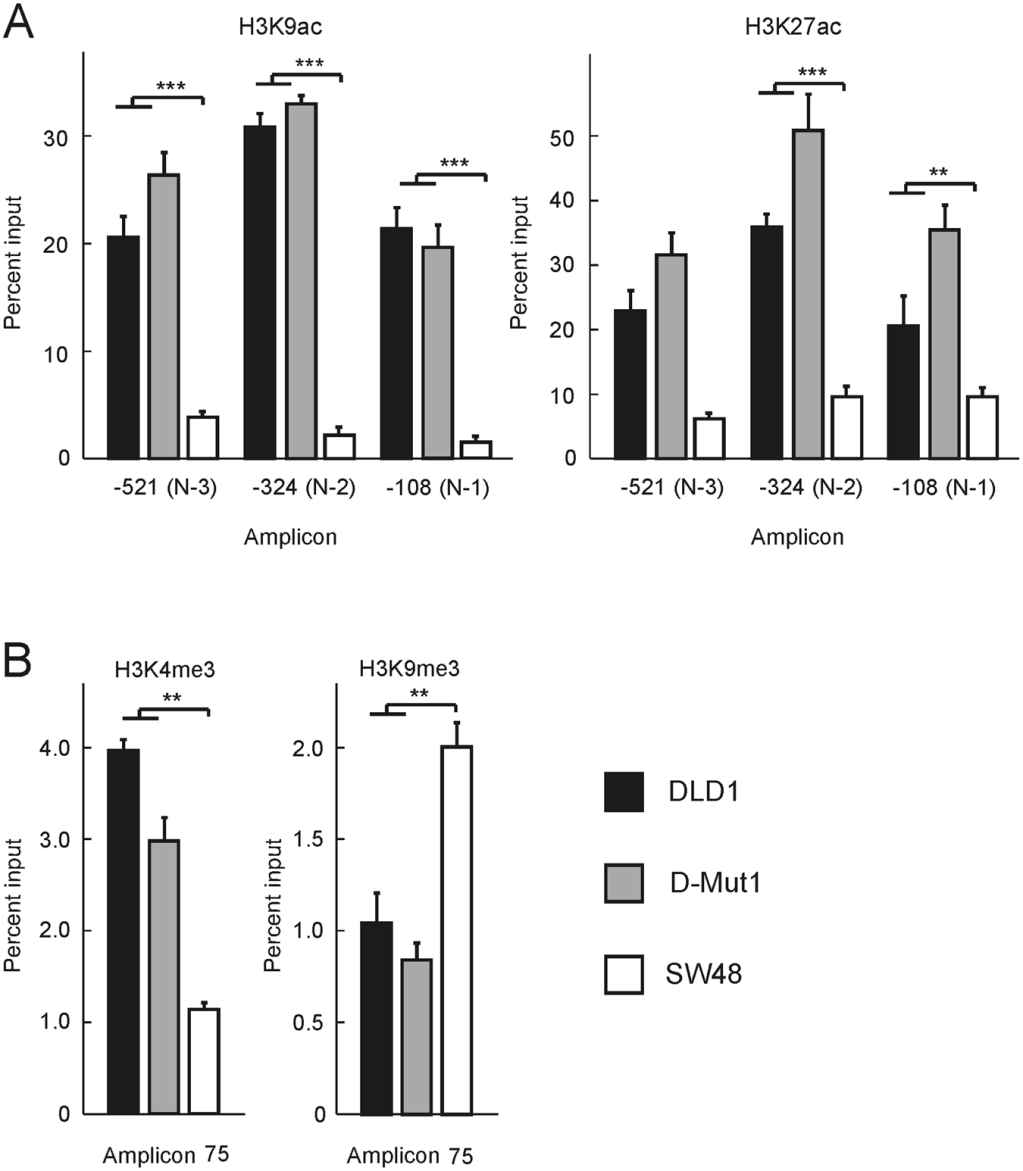
Epigenetic modification of histones in the promoter and proximal coding region of the *ZNF518B* gene. The experiments were carried out at single-nucleosome level by using the Nuc-ChIP approach. (**A**) Acetylation marks in nucleosomes N − 3, N − 2 and N − 1. (**B**) Methylation marks at amplicon 75, which covers the 5′ part of nucleosome N + 1 in DLD1 and D-Mut1 cells and the 3′ part of the nucleosome covering the TSS in SW48 cells. Results were compared with the Kruskal-Wallis test. (**p < 0.005; ***p < 0.001).

Epigenetic modification of histones is commonly associated with transcriptional regulation. Therefore, the presence of some epigenetic marks in the three cell lines was next studied. The determination was carried out by Nuc-ChIP, which allows the localisation of histone modifications at single nucleosome level^11^. Acetylation at H3K9 and H3K27, associated with transcriptional activity^12,13^, is characteristic of promoter nucleosomes^10^. H3K4me3 and H3K9me3, which are usually associated, respectively, with active and repressed chromatin^12–15^, are preferentially found in the proximal transcribed region^10^. Therefore, Nuc-ChIP to quantify H3K9ac and H3K27ac was done at amplicons −521, −324 and −108, which cover, respectively, nucleosomes N − 3, N − 2 and N − 1; H3K4me3 and H3K9me3 were analysed at amplicon +75, which cover the first part of nucleosome N + 1 in DLD1 and D-Mut1 cells and the second part of the nucleosome occupying the TSS in SW48. Figure 7A shows that nucleosomes N − 3, N − 2 and N − 1 are enriched in H3K9ac and H3K27ac in DLD1 and D-Mut1 cells, while the marks are less abundant in SW48 cells. H3K4me3 is more abundant in nucleosome N + 1 from the cells in which the gene is transcribed. On the contrary the mark H3K9me3 is especially present in the nucleosome covering the TSS in SW48 cells (Fig. 7B).

**Figure 8:**
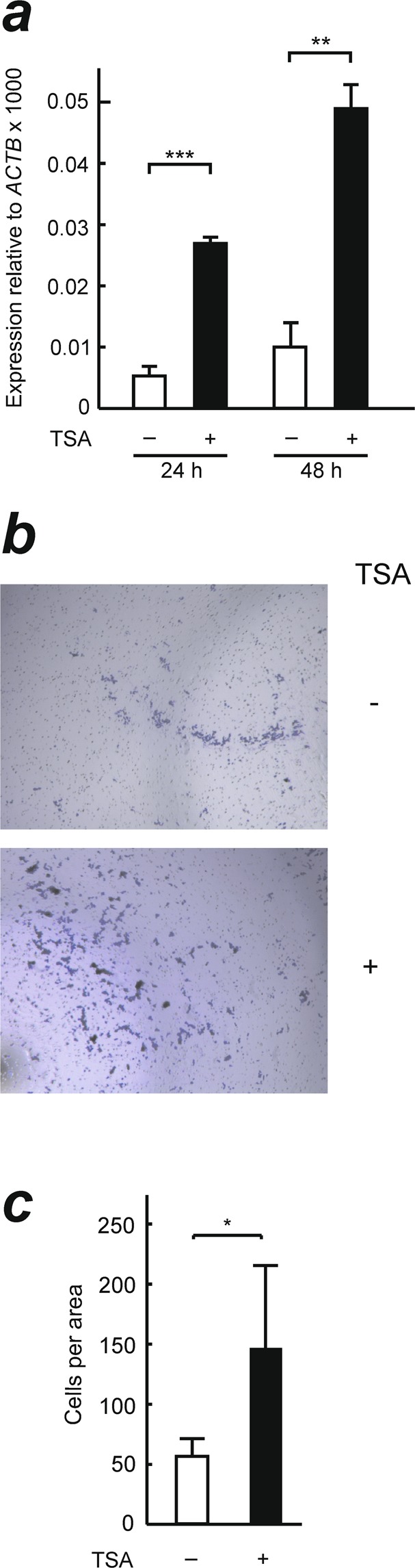
Effects of the inhibition of histone deacetylases on the expression of *ZNF518B* gene and in the migration of SW48 cells. (**a**) A culture of cells was treated either with trichostatin A (TSA) or with the solvent alone (DMSO) for 24 or 48 h and the level of *ZNF518B* expression relative to *ACTB* was determined by RT-qPCR as described in the text. (**b**) Representative images of SW48 cells migration determined by transwell analysis in the absence or presence of TSA for 24 h. (**c**) Quantification of cell migration. Two areas of three independent wells were counted and the six values averaged. Data were analysed with Student’s t-test. (**p < 0.005; ***p < 0.001).

To check that histone acetylation is actually involved in the regulation of *ZNF518B* transcription, we treated SW48 cells, in which the gene is not appreciably expressed, with trichostatin A (TSA), an inhibitor of histone deacetylases. The treated cells showed an enhancement in H3 acetylation (Supplementary Fig. S5), a significant time-dependent increase in *ZNF518B* expression (Fig. 8a) and a greater migration rate (Fig. 8b,c), a result compatible with the TSA-induced changes in *ZNF518B* expression. The experiments described under the present heading are compatible with the idea that the organisation of chromatin and its epigenetic marks are important factors in the regulation of *ZNF518B* transcription in the CRC cell lines studied.

## Discussion

The results described here, obtained from both a cDNA array of 43 tumour and 5 normal mucosa samples and from a local prospective cohort including 70 tumour and 69 adjacent non-tumour samples, show for the first time that the expression of *ZNF518B* may correlate with CRC. We have previously reported that the expression of *ZNF518B* isoforms 1 and 2 is roughly equivalent in HCT116, DLD1, D-Mut1 and DWT7m CRC cell lines^3^ and present results show that the levels of both isoforms are similar in CRC patients. In this paper we show that isoform 1 is up-regulated in a large cohort of patients and the analysis of isoform 2 is now being studied in our group. This issue may prove to be interesting, because isoform 2 mRNA is putatively translated to give a truncated product, consisting of the 75 N-terminal amino acids. Should this polypeptide actually occur, it may compete with the whole protein derived from isoform 1 mRNA in its interaction with G9A, because these truncated polypeptides from the N-terminus of the protein are still able to bind the histone methyltransferase^7^. Both ZNF518B isoforms might then play a functional role; isoform 1 as a zinc-finger containing transcriptional factor interacting with G9A, and isoform 2, devoid of the zinc-finger domains, competing with isoform 1. Interaction of isoform 1 with G9A results in the activation of the enzyme^7^, which is involved in invasiveness and metastasis in several cancer types^16,17^. We do not know whether isoform 2 would compete with isoform 1 in the binding to G9A. If it were, changes in the ratio of isoforms would represent an additional way to regulate the activity of the methyltransferase.

Present knocking-down experiments with *ZNF518B* siRNA showed that the expression of the gene does not influence cell growth. Nevertheless, the gene is clearly involved in cell migration and invasiveness, probably through type I collagen fibres. These fibres play a crucial role in cancer progression (for a review, see^18^), and they have been described as the “highways” for tumour cell migration^19^. It remains to be determined whether the effects of ZNF518B in invasiveness are mediated by the activation of G9A. It has been reported that G9A represses the expression of E-cadherin^17^ and the results of Fig. 5 show that the down-regulation of *ZNF518B* results in a significant increase of *CDH1* expression. Therefore, the suggestion can be made that the effects of *ZNF518B* on cell migration are related to the activation of the G9A methyltransferase.

The dysregulation of signalling pathways in cancer has been widely studied, as it often causes changes in gene expression that ultimately result in the development of malignancies. However, less attention has been paid to the involvement of chromatin structure and modifications in the regulation of particular genes involved in cancer. The importance of this issue cannot be disregarded, because the regulation of eukaryotic gene expression has to be considered within the landscape of chromatin. In this sense, the value of the experiments described here is reinforced, as they strongly suggest that the expression of *ZNF518B* is regulated by chromatin structure and epigenetic factors. The presence of a sequence-directed, positioned nucleosome covering the TSS in SW48 cells precludes the assembly of the pre-initiation complex resulting in transcriptional repression. On the contrary, a nucleosome-free region exists in *ZNF518B*-expressing cells around the TSS. Probably, the action of a chromatin-remodelling complex is responsible for these changes in chromatin structure and alterations in these remodelling machines have been described in many types of cancer^20^. Moreover, mutations in the ATPase subunit of SWI/SNF complex have been considered as drivers of cancer^21^.

Apart from chromatin organisation, epigenetic modification of histones is also involved in the regulation of *ZNF518B* expression. The nucleosomes N − 3, N − 2 and N − 1 of DLD1 and D-Mut1 cells, in which the gene is transcribed, are enriched in H3K9ac and H3K27ac, a signature of promoter nucleosomes in active genes^10^, whereas the level of these marks is significantly lower in SW48 cells. Our results prove that enhancing the level of H3 acetylation by TSA treatment results in a noticeable transcription of *ZNF518B* in SW48 cells, which, otherwise, express the gene to a negligible level. The methylation marks at N + 1 nucleosome are also compatible with their role in the regulation of gene expression. When these circumstances are considered together with the assumption that *ZNF518B* is involved in EMT, which, in turn, has been associated to modifications in the histone epigenetic marks^22^, the *ZNF518B* gene might be added to the list of epigenetic targets for cancer therapy. As TSA affects the expression of multiple genes in cancer cells^23^, a causal relationship between TSA treatment and increased migration of SW48 cells cannot be established from the experiments shown in Fig. 8. Anyway, it is clear that histone acetylation is implicated in the regulation of *ZNF518B* expression, which, in turn, is involved in cell migration and other pathogenic effects. Fortunately, several therapeutic approaches are being developed in this sense^20,24^ and it would be worth exploring these possibilities in the case of *ZNF518B* gene. Further work, based on larger patients’ cohorts, may decide its actual clinical value as a candidate for invasiveness prognosis in CRC, as well as its potentiality as a target for epigenetic drugs.

## Methods

### Human CRC samples

*ZNF518B* expression was first analysed inTissueScan CRC cDNA arrays (OriGene, HCRT101, Rockville, USA). Clinical samples were obtained from the INCLIVA biobank. 101 paraffin-embedded CRC paired samples from stages I-IV patients were used; their clinicopathological characteristics are given in Supplementary Table S1. Written informed consents were obtained from all the patients before surgery and all the procedures were carried out in accordance with the Declaration of Helsinki and other relevant guidelines. The study was approved by the Ethical Committee of the Hospital Clínico from the University of Valencia (Comité Ético de Investigación Clínica del Hospital Clinic Universitari de València, No. 2017/229).

### Cell culture

The human CRC cell lines SW48 (Horizon), HCT116 (ATCC CCL-247), DLD1 (ATCC CCL-221) and its isogenic derivative D-Mut1 (a gift from Dr B. Vogelstein), were grown as previously described^3^. To guarantee the continued quality of the cell lines used in this study, a short tandem repeat DNA profiling of the cells was performed. Genomic DNA was extracted at different steps of the study and Bioidentity (Elche, Spain), a biotechnology company of genetic analysis, monitored the identity of the cells. The analyses confirmed the identity of the cell lines, complying with international standards of authentication (ANSI/ATCC).

To inhibit histone deacetylases, cells were loaded in 6-well plates and treated with 0.25 μM TSA (Sigma). Controls were treated with the dimethylsulphoxide-containing solvent.

### *ZNF518B* knockdown

DLD1 and HCT116 cells were cultured in 6 well plates to 50–60% confluence and transfected with 20 nM of either scrambled siRNA (Qiagen 1027280), *ZNF518B* siRNAs (Qiagen SI04131015) or (Qiagen SI04284805). Lipofectamine RNAi Max (#1875252 Invitrogen) was used according to the instructions of the manufacturer. The cells were then incubated in CO_2_ atmosphere for several times at 37 °C. The efficiency of knockdown was checked by RT-qPCR and western blotting.

### Cell proliferation assay

Cell proliferation was measured by the 3-(4,5-dimethylthiazol-2-yl)-2,5-dip-henyltetrazolium bromide (MTT) assay. After 24 h of transfecting the cells with scrambled siRNA, or with the *ZNF518B* siRNAs, 3,000 cells per well were seeded in quadruplicate in 96-well plates and 10 μl of a 5 mg/ml solution of MTT in phosphate buffered saline (PBS) were added to each well containing 100 μl of culture medium at several times after seeding. The spectrophotometric determination of proliferation was done following the recommendations of the manufacturer.

### Cell migration and invasion assays

Cell migration and invasion assays were carried out in 12 well plates (Corning, Falcon, Cell Culture Inserts, with 8 μm pore size). For invasion assays, 50 μl matrigel (BME 2 RGF PathClear, Sigma-Aldrich) were loaded in the upper chamber prior to the introduction of the cells. For both migration and invasion assays, 10^5^ cells were seeded in 100 μl foetal bovine serum-free McCoy medium in the upper chamber and the lower chamber contained McCoy medium either with foetal bovine serum or without it as a negative control. The assay was stopped 24 h (for migration) or 48 h (for invasion) after seeding. Cells adhering to the lower surface were fixed with 70% methanol and stained with 0.2% crystal violet, counted under microscope in 6 different areas and the counting values were averaged. For wound healing assays, the Radius 24 cell migration assay kit (Cell Biolabs, Inc. CBA-125) was used. Every well was loaded with 8,000 cells and, after 24 h, the central gel layer was removed following the manufacturer’s instructions and the migration was checked by photographing the plates at 24, 48 and 72 h.

### Colony formation assay

Transfected cells were seeded in 6 well-plates at a density of 150 cells per well. The growing of cells were microscopically followed every day and after two weeks, the medium was withdrawn, the cells washed, fixed and stained as described above for migration and invasion assays. The total number of colonies (approximately more than 50 cells) was counted.

### Cell adhesion assays

Collagen type I-coated 60 mm dishes (Corning BioCoat Collagen I Cellware) were loaded with 30,000 48 h-silenced cells suspended in McCoy’s medium and, after 30 min the medium was withdrawn, the dishes washed with PBS and the adhering cells were counted as above.

### Flow cytometry analysis of cell cycle distribution

To evaluate cell cycle changes related to *ZNF518B* knockdown, trypsinized cells after silencing for 72 h were incubated with a hypotonic propidium iodide solution at 4 °C for 12 h. The samples were analysed in a Gallios TM Beckman Coulter cytometer following the manufacturer’s protocol. The distribution of cells in different phases was calculated by using FloJo software (TOMY Digital Biology, Tokyo, Japan). Each experiment was repeated at least three times.

### Analysis of chromatin structure and histone epigenetic modifications

Nucleosome occupancy was determined by the micrococcal nuclease protection assay^10,25^, using tiled amplicons of about 100 bp in size. The primers used are given in Supplementary Table S2. The experimental results were compared with the output of the sequence-based prediction of positioning carried out using the NuPoP software tool^26^. Epigenetic modifications of histones were studied at nucleosomal level by Nuc-ChIP^10,25,27^. The following antibodies were used: anti-H3K9me3 (Abcam, Cambridge, UK, ab-8898); anti-H3K4me3 (Abcam, ab-8580); anti H3K9ac (Abcam, ab-4441); anti H3K27ac (Abcam, ab-4729).

### Western blot analyses

Western blot analyses were carried out essentially as previously described^28^. Briefly, cultured cells were extracted with RIPA buffer (50 mM Tris-HCl pH 7.5, 150 mM NaCl, 1% Triton X-100, 0.1% SDS, 0.5% deoxycholic acid sodium salt (w/v)) supplemented with 2 μl/ml protease inhibitor cocktail (Sigma). Samples were sonicated and centrifuged at 13,000 × g for 30 min at 4 °C. Total protein was determined by a BCA protein assay kit (Thermo Scientific, Rockford, IL, USA). Nuclear fractions were obtained with the Nuclear Extract Kit (Active Motif #40010), following the manufacturer’s protocol. When ZNF518B protein is determined in nuclear SW48 cells extracts, electrophoresis was carried out in 6% polyacrylamide gels and the transfer was done overnight. Immunoblots were visualized using the ECL Western Blotting detection kit reagent (GE Healthcare) and the ImageQuant LAAS 400 system (Healthcare Bio-Sciences). The antibodies used were: anti-E-cadherin (CST, Cell Signaling Technology, Leiden, The Netherlands (24E10) #3195); anti-N-cadherin (CST, (D4R1H) #13116); anti-SNAIL (CST, (C15D3) #3879), anti-H3 (Merck-Millipore 07690), anti-acetylated proteins (Abcam ab-193), anti-ZNF518B (Sigma, HPA031216) and, as loading control, anti-GAPDH (Abcam ab-8245) or anti-HDAC1 (Santa Cruz, sc-8410). For a semi-quantitative determination of protein in the western blots, four grey values relative to the loading control were measured with ImageJ and averaged.

### RT-qPCR analysis of the expression of *ZNF518B*

The transcription level of the *ZNF518B* isoforms was determined by RT-qPCR using primers specific for each isoform as previously described^3^. The whole-gene expression was determined by RT-qPCR with the following primers: GGGCCTGAGGTTGTGAAACT (forward) and AAAACCGTGGCAAGTCCCAT (reverse), which amplify a region common to the major isoforms; only two isoforms expressed to a negligible level^3^ are not amplified. To analyse the expression of SNAIL gene (*SNAI1*), the following primers were used: ACCACTATGCCGCGCTCTT (forward) and GGTCGTAGGGCTGCTGGAA (reverse). The primers for E-cadherin gene (*CDH1*) were: GTCAGTTCAGACTCCAGCCC (forward) and AAATTCACTCTGCCCAGGACG (reverse). The β-actin gene (*ACTB*) was used for normalisation, with the primers: GTGCTATCCCTGTACGCCTC (forward) and GAGGGCATACCCCTCGTAGA (reverse). The relative gene expression was calculated by qPCR and normalized to actin expression according to the standard ΔΔC_T_ comparative method^29^.

### Statistical analyses

Quantitative values are expressed as mean ± SD of at least three determinations. Data in the different PCR determinations were compared by two-tailed t-test. The data given as box plots were compared by using either the Mann-Whitney or the Kruskal-Wallis test (for uni- or multivariate analyses, respectively). Differences were considered significant at p < 0.05.

## Supplementary information

Supplementary Material

## References

[1] Arnold M (2017). Global patterns and trends in colorectal cancer incidence and mortality. Gut.

[2] Christofori G (2006). New signals from the invasive front. Nature.

[3] Riffo-Campos Á (2016). In silico RNA-seq and experimental analyses reveal the differential expression and splicing of EPDR1 and ZNF518B genes in relation to KRAS mutations in colorectal cancer cells. Oncol. Rep..

[4] Jin T (2015). Genetic variations in the CLNK gene and ZNF518B gene are associated with gout in case-control sample sets. Rheumatol Int..

[5] Zhang XY, Geng TT, Liu LJ, Yuan DY, Feng T (2015). SLC2A9 and ZNF518B polymorphisms correlate with gout-related metabolic indices in Chinese Tibetan populations. Genet. Mol. Res..

[6] Köttgen A (2013). Genome-wide association analyses identify 18 new loci associated with serum urate concentrations. Nat. Genet..

[7] Maier VK (2015). Functional proteomic analysis of repressive histone methyltransferase complexes reveals ZNF518B as a G9A regulator. Mol. Cell. Proteomics.

[8] Shankar SR (2013). G9a, a multipotent regulator of gene expression. Epigenetics.

[9] Zhang J (2015). Down-regulation of G9a triggers DNA damage response and inhibits colorectal cancer cells proliferation. Oncotarget.

[10] Riffo-Campos ÁL (2015). Nucleosome-specific, time-dependent changes in histone modifications during activation of the early growth response 1 (*Egr1*) gene. J. Biol. Chem..

[11] Castillo J, López-Rodas G, Franco L (2017). Histone post-translational modifications and nucleosome organisation in transcriptional regulation: Some open questions. Adv. Exp. Med. Biol..

[12] Peterson CL, Laniel M-A (2004). Histones and histone modifications. Curr. Biol..

[13] Kimura H (2013). Histone modifications for human epigenome analysis. J. Hum. Genet..

[14] Barski A (2007). High-resolution profiling of histone methylations in the human genome. Cell.

[15] Wang Z (2008). Combinatorial patterns of histone acetylations and methylations in the human genome. Nat. Genet..

[16] Liu S (2015). G9a is essential for EMT-mediated metastasis and maintenance of cancer stem cell-like characters in head and neck squamous cell carcinoma. Oncotarget.

[17] Hsiao SM (2015). The H3K9 methyltransferase G9a represses E-cadherin and is associated with myometrial invasion in endometrial cancer. Ann. Surg. Oncol..

[18] Venning FA, Wullkopf L, Erler JT (2015). Targeting ECM disrupts cancer progression. Front. Oncol..

[19] Egeblad M, Rasch MG, Weaver VM (2010). Dynamic interplay between the collagen scaffold and tumor evolution. Curr. Opin. Cell Biol..

[20] Dawson MA (2017). The cancer epigenome: Concepts, challenges, and therapeutic opportunities. Science (80-.)..

[21] Wu Q (2017). The BRG1 ATPase of human SWI/SNF chromatin remodeling enzymes as a driver of cancer. Epigenomics.

[22] Tam WL, Weinberg RA (2013). The epigenetics of epithelial-mesenchymal plasticity in cancer. Nat. Med..

[23] Shin, S., Kim, M., Lee, S. J., Park, K. S. & Lee, C. H. Trichostatin a sensitizes hepatocellular carcinoma cells to enhanced NK cell-mediated killing by regulating immune-related genes. *Cancer Genomics and Proteomics* (2017). 10.21873/cgp.20045 PMC5611521 28871002

[24] Balch C, Ramapuram JB, Tiwari AK (2017). The epigenomics of embryonic pathway signaling in colorectal cancer. Front. Pharmacol..

[25] Sacilotto N, Espert A, Castillo J, Franco L, López-Rodas G (2011). Epigenetic transcriptional regulation of the growth arrest-specific gene 1 (*Gas1*) in hepatic cell proliferation at mononucleosomal resolution. PLoS One.

[26] Xi L (2010). Predicting nucleosome positioning using a duration Hidden Markov Model. BMC Bioinformatics.

[27] Riffo-Campos ÁL (2018). Role of epigenetic factors in the selection of the alternative splicing isoforms of human KRAS in colorectal cancer cell lines. Oncotarget.

[28] Sacilotto Natalia, Castillo Josefa, Riffo-Campos Ángela L., Flores Juana M., Hibbitt Olivia, Wade-Martins Richard, López Carlos, Rodrigo M. Isabel, Franco Luis, López-Rodas Gerardo (2015). Growth Arrest Specific 1 (Gas1) Gene Overexpression in Liver Reduces the In Vivo Progression of Murine Hepatocellular Carcinoma and Partially Restores Gene Expression Levels. PLOS ONE.

[29] Livak KJ, Schmittgen TD (2001). Analysis of relative gene expression data using real-time quantitative PCR and the 2^−ΔΔC^_T_ method. Methods.

