# Supplementary material for: ZNF518B gene up-regulation promotes dissemination of tumour cells and is governed by epigenetic mechanisms in colorectal cancer
Source: Sci Rep. 2019 Jun 27;9:9339. doi: 10.1038/s41598-019-45411-9 (PMC6597559; doi:10.1038/s41598-019-45411-9)
Supplement: Supplementary file 1 — Supplementary Material [file 41598_2019_45411_MOESM1_ESM.pdf]

ZNF518B gene up-regulation promotes dissemination of tumour cells and is governed by epigenetic mechanisms in colorectal cancer

Francisco Gimeno-Valiente, Ángela L. Riffo-Campos, Azahara Vallet-Sánchez, Sofía Siscar-Lewin, Valentina Gambardella, Noelia Tarazona, Andrés Cervantes, Luis Franco, Josefa Castillo and Gerardo López-Rodas

Supplementary Material

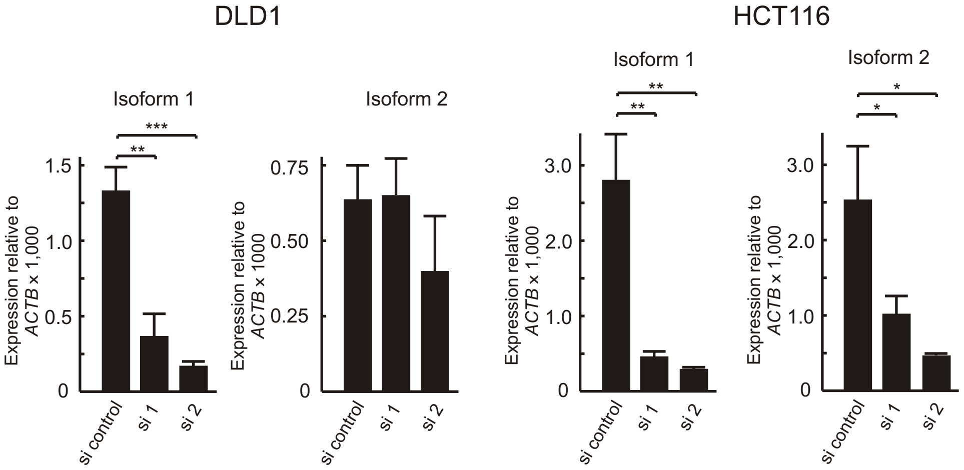

Figure S1. Efficiency of individual siRNAs on the expression of *ZNF518B* isoforms.The knocking down level was quantified in DLD1 and HCT116 cell lines by RT-qPCR. Three PCR determinations of two independent experiments (total n = 6) were done. In the figures si control stands for scrambled siRNAs; si 1 is Qiagen #SI04284805 and si 2 is Qiagen #SI04131015. The quantitative results were analysed by Student's t test: *, $p < 0.05$; **, $p < 0.01$; ***, $p < 0.0001$.

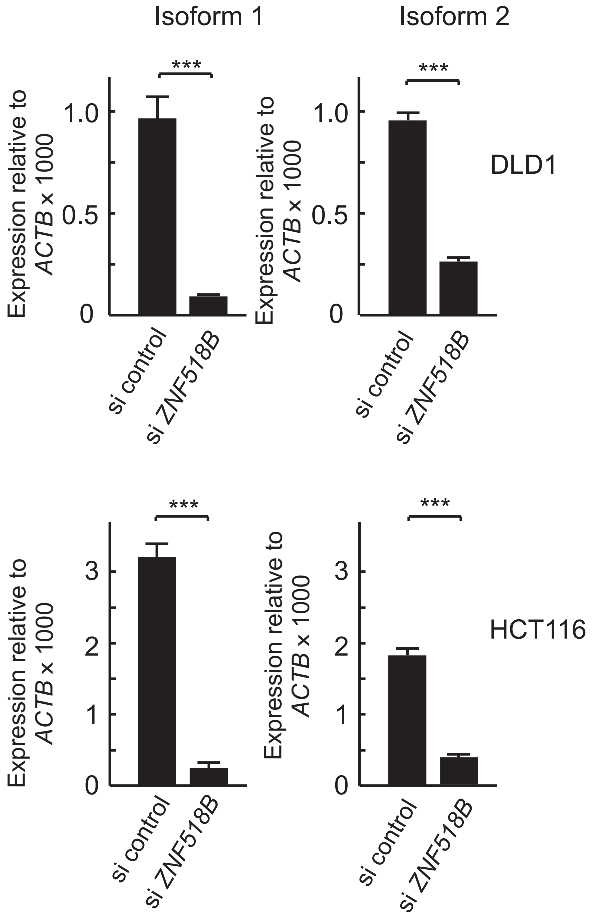

Figure S2. Efficiency of knocking-down for isoforms 1 and 2 of *ZNF518B* in DLD1 and HCT116 cells after 96 h of transfection with scrambled siRNAs (si control) or with the mixture of siRNAs (si*ZNF518B*). Results were analysed by Student's t-test. (***, $p < 0.001$).

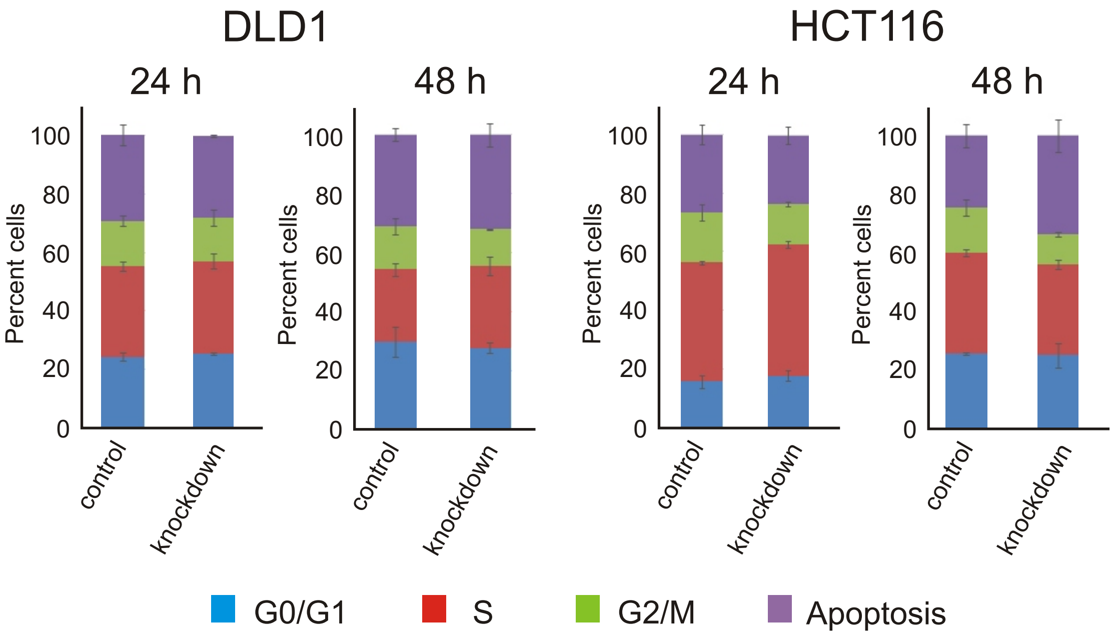

Figure S3. Effects of knocking-down the *ZNF518B* gene on the cell cycle and apoptosis of DLD1 and HCT116 CRC cells.

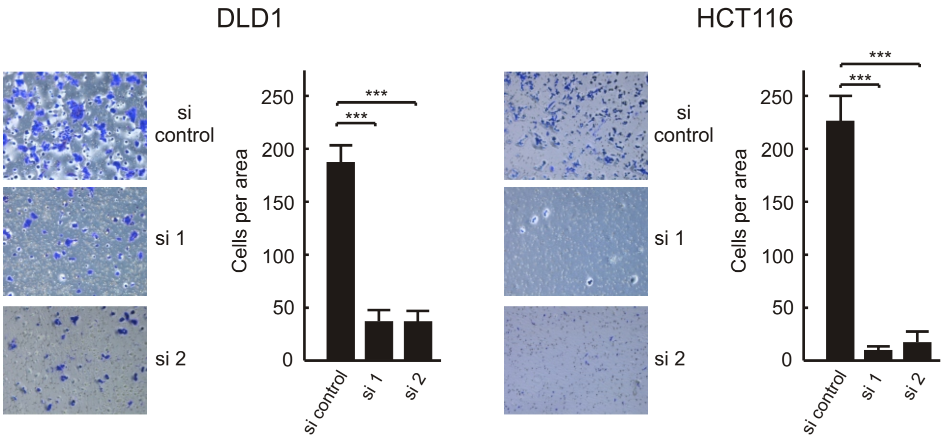

Figure S4. Effects of individual *ZNF518B* siRNAs on the supression of migration of DLD1 and HCT116 cells by. The assay was done in transwell chambers; representative images and the average results of cell counting in 9 wells were shown. In the figures si control stands for scrambled siRNAs; si 1 is Qiagen #SI04284805 and si 2 is Qiagen #SI04131015. The quantitative results were analysed by Student's t test: ***, $p < 0.0001$.

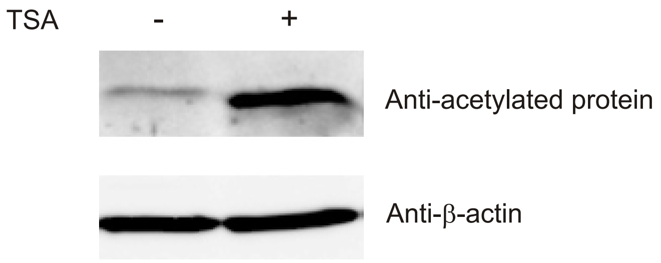

Figure S5. Western blots showing the effects of the use of HDAC inhibitor on the level of H3 acetylation in Sw48 cells. β-actin was used as loading control.

Supplementary Table S1: Clinicopathological characteristics of the patients' cohort (n=101).

**Patients' characteristics (n)**		
Median Age		73
Age range		37-96
Sex	Male	61
	Female	40
AJCC Stage	I	12
	II	35
	III	35
	IV	19
Vascular invasion	Yes	35
	No	56
	NR*	10
Peritoneal invasion	Yes	30
	No	61
	NR*	10

NR: Not reported.

Supplementary Table S2. Primers used for nuclease protection assay and Nuc-ChIP experiments.

Amplicon	Primer sequence		Size (bp)
	Forward	Reverse	
-1012	CAACTCCGCTTCTCCGTGT	CATCACCTTCCTGTTGGCGG	110
-915	GTGATGGAAACCAGCCTTGC	CAGCGTCACTGGGAAGTACA	96
-845	ACTGTACTTCCCAGTGACGC	GCACAATCAGTGACTCCCCA	87
-775	GAGTCACTGATTGTGCGGGA	TACGGTCCGGAAGGAAGACA	89
-715	TATGTCTTCCTTCCGGACCGT	TTCGGTGCTATTTTGGGCGA	71
-651	GTTGGCTCGCCCAAAATAGC	AGGGTCAGAGGGGACAATGA	110
-580	CATTGTCCCCTCTGACCCTC	GGGAAGGTCGTATGTGAGGC	70
-521	CTCACATACGACCTTCCCCG	TCTTAGCTGCTGGACCTTGG	84
-453	TTTCTCGCCAAGGTCCAGC	TCGTCTTCTTGGAGAGCTTCAG	107
-385	ACTGAAGCTCTCCAAGAAGACG	GTGGGTGAGTGAAAGCGAGA	74
-324	TCTCGCTTTCACTCACCCAC	CCTGCCTTGTACCGGGTC	88
-249	GACCCGGTACAAGGCAGG	AGCGAGGTCCACATTTAGCC	98
-165	GGCTAAATGTGGACCTCGCT	TTCTAAGCTGTCCAGAGCGG	109
-108	TCTGAGGCTCCCCGCTCT	CTGAGGTCTCTAAGGCTGCG	67
-80	CCGCTCTGGACAGCTTAGAA	GCGTGCGCAATGTGAAGC	102
-27	TCAGGCGCCGGCCTCGCTGGAG	CGCCGCGCCCGCTGTAGGTC	104
-16	CGCCGCTTCACATTGCG	GCCCGCTGTAGGTCCCTA	70
+6	GCCGCCGCTTCACATTGC	GGCGAAGGGGCGTCTACA	117
+79	CGGTGTAGACGCCCCTTC	CCTCCCGCTCAGCTACTTAC	71
+111	GACCTGGCACCCGAATC	CGCTTCGGTCACGTACAC	86
+120	TGCCGGGTAAGTAGCTGAG	GCGCTTCGGTCACGTACAC	65
+156	GAGGGCCCCCGGATCCGC	GTCAGGCCCCCGCCCCGC	118
+199	TGACCGAAGCGCCAGC	GACGCCAGCCCTCAGC	119
+272	CGTGCCCGCGCTGAGGGC	GCTGGTGGGCGTGGGGCA	78

The amplicons are identified after the position of their centre relative to the transcription start site
